# Smart & Green: An Internet-of-Things Framework for Smart Irrigation

**DOI:** 10.3390/s20010190

**Published:** 2019-12-29

**Authors:** Nidia G. S. Campos, Atslands R. Rocha, Rubens Gondim, Ticiana L. Coelho da Silva, Danielo G. Gomes

**Affiliations:** 1 Grupo de Redes de Computadores, Engenharia de Software e Sistemas (GREat), Departamento de Engenharia de Teleinformática, Centro de Tecnologia, Campus do Pici, Avenida Mister Hull, s/n, Bloco 942-A, Fortaleza 60.455-760, CE, Brazil; atslands@ufc.br (A.R.R.); danielo@ufc.br (D.G.G.); 2 Departamento de Telematica, Instituto Federal do Ceara, Campus Fortaleza, Avenida Treze de Maio, 2081, Benfica, Fortaleza 60.040-531, CE, Brazil; 3 Embrapa Agroindustria Tropical, Rua Dra. Sara Mesquita, 2270, Planalto do Pici, Fortaleza 60511-110, CE, Brazil; rubens.gondim@embrapa.br; 4 Instituto UFC Virtual, Universidade Federal do Ceará, Av. Humberto Monte, s/n, bloco 901, 1° andar. CEP, Fortaleza 60.440-554, CE, Brazil; ticianalc@ufc.br

**Keywords:** IoT, smart agriculture, soil moisture prediction

## Abstract

Irrigation is one of the most water-intensive agricultural activities in the world, which has been increasing over time. Choosing an optimal irrigation management plan depends on having available data in the monitoring field. A smart agriculture system gathers data from several sources; however, the data are not guaranteed to be free of discrepant values (i.e., outliers), which can damage the precision of irrigation management. Furthermore, data from different sources must fit into the same temporal window required for irrigation management and the data preprocessing must be dynamic and automatic to benefit users of the irrigation management plan. In this paper, we propose the Smart&Green framework to offer services for smart irrigation, such as data monitoring, preprocessing, fusion, synchronization, storage, and irrigation management enriched by the prediction of soil moisture. Outlier removal techniques allow for more precise irrigation management. For fields without soil moisture sensors, the prediction model estimates the matric potential using weather, crop, and irrigation information. We apply the predicted matric potential approach to the Van Genutchen model to determine the moisture used in an irrigation management scheme. We can save, on average, between 56.4% and 90% of the irrigation water needed by applying the Zscore, MZscore and Chauvenet outlier removal techniques to the predicted data.

## 1. Introduction

Agricultural activities presently use 70% of the withdrawn freshwater in the world [1]. Therefore, it is of fundamental importance that we apply irrigation management, especially in semiarid regions with a scarcity of rainfall. Irrigation management is a part of precision agriculture, in which the correct amount of water artificially delivered to a field to satisfy the crop needs and the real production of the users is analyzed [2].

The irrigation must supply the crop water needs at different developmental stages in a given local. Irrigation management is used to detect when to irrigate, the amount of water needed, and the irrigation frequency, based on the monitoring of crop evapotranspiration and soil moisture conditions. Crop evapotranspiration measures the water consumption by crops, according to the developmental stage of the crop and the weather conditions [3]. The soil moisture impacts the amount of irrigation water given to the crop, as irrigation management takes into account the level of water retention in the soil. Therefore, precision agriculture can reduce water consumption in irrigation by considering the groundwater available to the crop [2].

The choice of the best irrigation management plan depends on the data available from the monitoring field. A smart agriculture system gathers and processes data for irrigation management from several sources using computer science and information technology [4]. Users can provide the features of different types of crop, soil, and irrigation system, as well as the readings of analog tensiometers. Tensiometers are soil sensors that detect the matric potential, which are used to estimate the moisture at different depths and monitoring points in a field. Automatic weather stations [5] can provide public data through the Internet. Moreover, the field may have sensors and actuators which can interact with each other as objects of the Internet of Things (IoT) in order to provide services transparently to the users [6]. Such services are related to irrigation management for monitoring (i.e., water, soil, and air) and prediction (weather and soil conditions).

However, data are not free of discrepant values (i.e., outliers), which can negatively affect the precision of irrigation management. Furthermore, data from different sources must fit into the same temporal window required for the irrigation management and the data preprocessing must be dynamic and automatic for researchers, engineers, and owners or farmers to benefit from the irrigation management plan. Data fusion is required to improve the quality of soil data in the case where several sources provide the same type of data (e.g., moisture) from the field [7]. Data fusion also informs the decision whether to start irrigation or not in irrigation management that makes use of other types of data (such as weather or crop data).

In this paper, we propose the Smart&Green framework to offer services for smart irrigation, such as data monitoring and control, preprocessing, fusion, synchronization, storage, and irrigation management enriched by the prediction of soil moisture. The Smart&Green services are modular components which facilitate the reuse and customization of code, which are challenges/limitations to applying IoT in the smart agriculture context [8]. Our framework allows users to set the data sources: The nearest weather station, the moisture sensors (analog or digital), the type of crop, and the features of the irrigation system in the monitored field. Therefore, Smart&Green also contributes to the solution of another challenge in applying IoT for the purpose of smart agriculture: The integration of the actual infrastructure of the users (i.e., devices, machines, and software) [9].

Smart&Green recommends the best irrigation management plan, according to the configuration of the monitored field. The framework also synchronizes weather and soil data, as well as the crop stage for the chosen irrigation management plan. The Fusion service allows for the definition of outlier removal criteria for the weather and soil data. The fusion service also applies the outlier removal techniques Chauvenet, Z-Score, Modified Z-Score, and Generalized ESD before data aggregation, in order to obtain general information about the soil conditions of a field. Our results show that it makes irrigation management more precise, therefore saving water.

For fields without soil moisture sensors, the prediction module estimates the soil moisture to make the amount of water irrigated more precise. In this work, we propose a computational model to predict the matric potential based on weather data available at public stations [5], crop information, and the amount of irrigation water used. As another contribution, we provide to the scientific community a public data set containing the matric potential and irrigation data for cashew cultivation in an experimental field. The matric potential is the critical variable in measuring the soil moisture.

The computational model reduces the cost of equipment and energy, as the model allows farms to estimate the moisture without soil sensors for the same soil type. We evaluate the performance of a set of popular machine learning techniques which have been widely applied to solve regression problems. We measure the quality of the models without feature selection using the RMSE (Root Mean Squared Error) and the MAE (Mean Absolute Error) as metrics. Our results show that the combination of predicted data and the outlier removal techniques can save an average between 56.4% and 90% of irrigation water, estimated by water balance management.

The rest of the paper is structured as follows: In Section 2, a literary review of IoT platforms for Smart Agriculture is presented. Section 3 describes the structure and services of the Smart&Green framework. Section 4 relates the materials and method of the implementation of Smart&Green, especially for the soil moisture prediction evaluation. The results are given in Section 5, and we discuss our contributions in Section 6. Appendix A gives details about irrigation management using Smart&Green, and we formalize the problem of soil moisture prediction in Appendix B.

## 2. Related Work

We carried out a systematic literature review to find the works which have answered the following research questions:

1. What software exists for agricultural management that automates the process of gathering, preprocessing, fusing, and synchronizing the data used in irrigation management?
2. Does the software implement the well-known irrigation management approaches of matric potential (Equation (A2)) and water balance (Equation (A3))?
3. Does the software forecast the soil moisture?
4. Can users configure the software using information about their crops, irrigation system, soil sensors, and weather stations close to the monitored field?

Most of the discovered papers have partially answered the research questions above. To our best knowledge, there have been almost no Internet of Things (IoT) platforms introduced that provide the services required for smart irrigation, such as the gathering, preprocessing, synchronization, and fusion of data; the planning and execution of irrigation management; or soil moisture prediction.

Several studies have only addressed the gathering and visualization of weather and soil data (Section 2.1). Some works focused on data preprocessing by applying outlier detection algorithms (Section 2.2). Some approaches used specific methods to decide when to irrigate, whereas others have implemented water balance and matric potential (Section 2.3). Soil moisture prediction using machine learning techniques applied to crop data, satellite images, and irrigation management information has been investigated (Section 2.4). In addition, IoT platforms have been studied, which should be flexible enough to allow users to configure them for different monitoring cases in smart agriculture (Section 2.5).

### 2.1. Data Gathering

In general, most of the sensor nodes used to monitor soil data (such as temperature, humidity, and matric potential) and weather data (such as temperature and relative humidity) are operated using open, low-cost hardware platforms such as the Arduino [10,11,12,13,14] or Raspberry Pi [15,16,17]. Zigbee [11,17,18,19], LoRa [16], Wifi [20], Bluetooth [14], GSM [12], and GPRS [21] are the predominant wireless technologies used.

Our proposal allows users choose between the CoAP [22] and MQTT [23] protocols for sending data to Smart&Green framework storage and later irrigation management. For devices that we can not turn into a sensor node, such as analog tensiometers, our framework has a mobile application with which users can gather data manually. The framework also allows users to choose, as a weather data source, the Brazilian automatic weather station nearest to the monitored field, from which data can be gathered via HTTP.

### 2.2. Data Preprocessing

Other approaches have processed soil data to detect outliers [24,25] and treat data using noise filtering techniques [26]. One system applied an EKF (Extended Kalman Filter) to the soil moisture data before the execution of irrigation management, in order to avoid the actuators inappropriately starting the irrigation [27].

The pattern recognition of water consumption by a crop radicular system, presented in [28], is an outlier detection technique based on a time-series analysis of soil moisture gathered at several depths before, during, and after irrigation. This technique allows for the identification of sensors with poor operation, which indicates the need for calibration or change.

Our proposed framework allows users to define criteria for outlier removal for each type of weather and soil data used in irrigation management. Our framework also gives the option to execute such algorithms as Chauvenet, Z-Score, Modified Z-Score, and Generalized ESD (Extreme Studentized Deviation) to remove outliers and fuse soil moisture data, in the case when a field has several soil sensors monitoring at different depths.

### 2.3. Irrigation Management

Other works have automatically started irrigation by implementing algorithms for analyzing the data gathered by a wireless sensor node. In one study, an algorithm based on decision trees used the soil temperature and moisture to determine the irrigation time and the needed amount of water [29]. Crisp rules (IF-THEN) were used to compare threshold values with the soil moisture gathered by sensors, in order to decide whether to turn on the sprinklers of an irrigation system, in [30,31].

Decision support platforms for the execution of traditional irrigation management, such as the matric potential (Equation (A2)) and water balance (Equation (A3)), have been introduced. These systems allow agronomists and farm owners to manage Internet of Things (IoT) components for field monitoring. The matric potential management uses soil data gathered by sensor nodes and images captured by unmanned aerial vehicles to correctly estimate the irrigation water need [32].

Web systems have been used for the planning and execution of irrigation management. Beyond the visualization of soil moisture and weather data gathered by wireless sensor nodes, these systems use crisp rules for data analysis and, when it is necessary, the systems send messages to their users in order to notify about irrigation times [33] or activate the irrigation system automatically [34,35]. In other approaches, the system takes the rainfall forecast (available on the Internet) into account when deciding whether to activate the irrigation system [36]. A wireless sensor network can also plan and execute irrigation management [37]. In this case, the sensor nodes gather and analyze soil moisture data and turn the irrigation system on until the moisture achieves the field capacity.

A SCADA (Supervisory Control and Data Acquisition) system can automatically carry out the data gathering, planning, and execution of the water balance management (see Equations (A3) and (A4)). The soil sensor nodes are also actuators, which control the irrigation and send data continually to a management system. The system receives data from a weather station on the farm to estimate the reference evapotranspiration $ET_{o}$. The system also utilizes the curve of the crop coefficient $k_{c}$, choosing its value according to the current developmental stage of the crop [38].

The water balance and matric potential approaches often must take data synchronization into account. Therefore, some applications have synchronized the data of previously registered crops, as well as soil and weather data, to precisely determine the irrigation water need for the current developmental stage of the crop [39,40,41,42].

The existing systems described in this section are not flexible enough to allow users to set the features of a monitored field, such as the type of crop, the type and number of soil sensors, and irrigation system parameters used. The systems usually work with a predefined configuration and infrastructure. Therefore, code reuse is impracticable for the irrigation management of agricultural fields which do not fit to the existing system configuration. Our proposal allows users to register relevant information for irrigation management: Different types of crops, soil features, irrigation systems, data sources (weather or soil), and outlier removal criteria. In this work, the framework plans the irrigation management using the matric potential (Equation (A2)) and water balance (Equation (A3)) approaches.

Furthermore, none of the systems could forecast the moisture levels in fields without soil sensors. Our proposal implements computational models to predict soil moisture based on weather data, crop information, and irrigation water need (IWN) (see Appendix B). In this work, we include the predicted data in the water balance management, obtaining between 56.4% and 90% of IWN savings over six months (Section 5.3).

### 2.4. Soil Moisture Prediction

In [43], the Linear Regression, Decision Tree, Random Forest, and Gradient Boosting with Regression Tree (GBRT) machine learning (ML) techniques were applied to weather and soil sensor data (moisture and drought stress) to predict the best irrigation plan for a jojoba crop. The GBRT model outperformed the others and, so, the decision support service incorporated it as a module of the system. A model based on support vector regression (SVR) and k-means ML techniques which used weather data and forecasts, as well the soil data (temperature and moisture), to forecast the soil moisture has also been investigated [44].

In [45], an integrated system to monitor drought in northern China using satellite images (NOAA, MODIS, and FY3A), weather stations, and soil data has been discussed. The drought forecasting used the matric potential at the depth of the crop root, the water balance model, depletion indices, and measurements of crop stress to estimate the soil moisture and the water quantity needed for an extended period.

**Figure A1:**
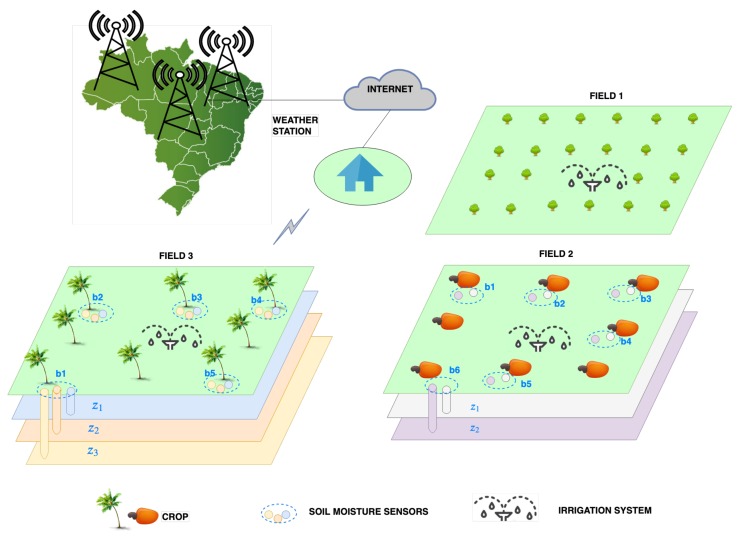
Different field configurations.

In this work, we apply weather data, crop information, and the amount of irrigation water used in a computational model, in order to estimate the daily matric potential of the most superficial soil layer (Appendix B), which signals when the crop needs irrigation [2]. We apply the predicted value in the Van Genutchen model (Equation (A1)) to obtain the soil moisture used in irrigation management (Equations (A2) and (A3)) for fields without soil moisture sensors (i.e., Field 1 of Figure A1).

We present two different approaches: Local and global prediction models. In the local method, we create a prediction model for each monitoring point in an experimental field (Section 4.2). In the global approach, we create a single model which can be generalized to any monitoring point. We have evaluated the performance of a set of popular ML techniques which have been widely applied to solve regression problems: Linear Regression [46], Decision Stump [47], M5 Model Trees and Rules [48,49], Random Tree, Random Forest [50], RepTree, and GBRT [51,52]. Our results show that GBRT outperforms the other evaluated techniques in both approaches.

### 2.5. IoT Platforms for Smart Agriculture

SWAMP (Smart Water Management Platform), introduced in [53], is one of the related methods which is most similar to our proposal. SWAMP has components to allow for the implementation of different IoT applications for irrigation management, based on the crop and soil moisture approaches. Users can customize the services for the gathering (MQTT or LoRa), processing, and synchronization of data with different types of crops, weather, and country. Therefore, different field configurations can reuse these services for data analysis and storage, highlighting the excellent flexibility of SWAMP. However, their techniques for the processing and the fusion of data have not been detailed, and no application or service for the prediction of soil moisture has been provided.

The Agro-IoT framework, introduced in [54], is also related to our proposal, which provides users real-time data gathering, aggregation (fusion), and analysis in the context of smart farming. Agro-IoT reuses several components to implement IoT applications, according to the needs of the monitored field. Agro-IoT provides similar services to our proposed method; that is, it also provides the management of devices (i.e., registration of sensors and actuators) and event detection through data analysis. However, it does not contain modules for data synchronization and outlier removal or soil moisture prediction.

## 3. Proposal

**Figure 1:**
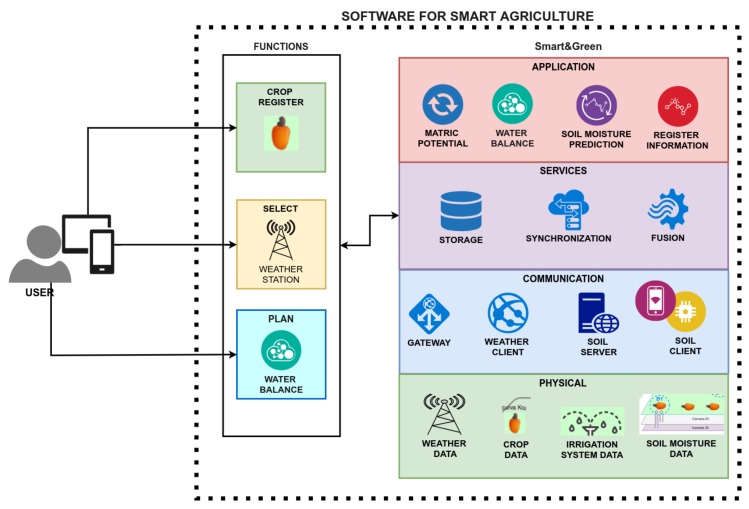
Conceptual architecture of the Smart&Green IoT Framework for Smart Agriculture.

In this paper, we propose Smart&Green: An Internet of Things (IoT) framework for the smart agriculture domain. A framework consists of “*a specific implementation of a skeleton of infrastructure used for the conception of a work*” [55]. Work is any activity performed by users or software. In this proposal, the work consists of the planning of irrigation management (Appendix A). In fields where there are no soil moisture sensors, a computational model can estimate the soil moisture for the same soil type using weather, crop, and irrigation data. Agronomists, researchers, and farmers conduct irrigation management through software which uses Smart&Green. Figure 1 presents the conceptual architecture of Smart&Green, divided into four layers: Application, Services, Communication, and Physical.

In software engineering, a framework can also be “*a mini architecture that can be reused and that provides behavior and a generic structure for a family of software abstractions in a context that specifies the collaboration and use of them into a given domain*” [56]. Therefore, given the smart agriculture domain and the context of smart irrigation, the Smart&Green framework specifies the structure of a set of concrete and abstract classes to implement modules for the gathering, storage, synchronization, and fusion of data, as well as mathematical models (Appendix A) which describe irrigation management. Irrigation management determines the behaviors of the classes and modules. As a mini architecture, the proposed framework can be reused with different smart agriculture software with the generic functionalities of irrigation management.

### 3.1. Application Layer

The application layer provides facilities for the automatization of irrigation management (Appendix A) and soil moisture prediction. Through this layer, users of an agriculture management system can insert information about the farm infrastructure: The types of crops and irrigation system to each monitored field, the monitored soil layers, and the weather stations closest to the farm.

#### 3.1.1. Irrigation Management Automatization

The Matric Potential and Water Balance modules implement well-known irrigation management methods (Appendix A). These modules use information registered by users as well as that from weather and soil data sources. Smart&Green selects the best irrigation management based on the data available. The principal functions of the Register Module are as follows:

1. *User register*: Smart&Green framework allows two types of users: Specialist and regular. Specialist users can provide agronomic information, such as different types of crops, soil, and irrigation system features. They can also register outlier removal criteria for weather and soil data and choose algorithms for the fusion service. Regular users can register farms and fields.
2. *Farm Register*: Users provide information about the farm, such as address and geographical co-ordinates (i.e., latitude and altitude) for the Smart&Green framework, and select the weather station closest to the farm.
3. *Crop register*: A specialist user can create types of crops using information such as a description, the curve of the crop coefficient, and the critical moisture condition.
4. *System Irrigation Register*: A specialist user inserts the type of irrigation systems, such as “micro-sprinkler”.
5. *Weather Station Register*: Smart&Green automatically selects the weather station closest to the farm using the geographical co-ordinates. Users can confirm this or choose another one.
6. *Soil Sensor Register*: The user can insert the types of soil sensors used. Smart&Green already has analog and digital tensiometers.
7. *Field Register*: Users set the field configuration features (Figure A1), such as the type of crop, irrigation system, soil, and if there are soil monitoring points. The developmental stage of the crop represents the number of days since the initial cultivation. Effective precipitation (mm/h) and efficiency of the irrigation system are necessary for irrigation management, in order to compute the irrigation time. In the case of monitoring points, users can register the type of soil moisture sensor (analog or digital) and the depth *z* monitored for each monitoring point in the field.
8. *Field Communication register*: In the case where the monitored fields have sensor nodes that automatically gather soil data, users can define the type of communication to send the data to the framework. Users set the IP address and specific configuration (CoAP or MQTT).
9. *Outlier Removal Criteria Register*: Specialist users can create a threshold for minimum and maximum values of each type of weather and soil moisture data used in irrigation management.

The Smart&Green framework suggests an irrigation management plan, according to the data of a registered field. For fields without soil moisture sensors (Field 1 of Figure A1), Smart&Green indicates the water balance without soil moisture data (i.e., without the term ΔA in Equation (A3)) or the water balance with the soil moisture data created by the soil moisture prediction module (Section 3.1.2). For fields with soil moisture sensors (see Fields 2 and 3 of Figure A1), the framework calculates the matric potential (Equation (A2)) or water balance using the soil moisture data.

#### 3.1.2. Soil Moisture Prediction

In this work, we address a large field (over 7500 m${}^{2}$) to produce one type of crop on a large scale, in which the soil is monitored by a hundred tensiometers (i.e., soil moisture sensors) at three different depths. To investigate this scenario, we present the local and global approaches, which can be used to learn a prediction function $\hat{f}$ (Appendix B).

The local approach creates a different prediction function $\hat{f_{i}}$ for each tensiometer at the superficial soil layer of the field. For this, it uses the observations recorded by a tensiometer $b_{i}$ and a weather station ms, as well the water amount $w_{i}$ given to the crop and the crop coefficient $k_{c}$ related to the current life stage of the crop. The local approach defines the prediction $\hat{f}$ in terms of *n* different functions $\hat{f_{i}}$ for local predictions. However, if a field has a large number of tensiometers, a large number of distinctive prediction models must be trained.

The global approach creates only one prediction function $\hat{f_{i}}$ using the information from all tensiometers. Therefore, the global prediction model might not fit some individual tensiometers.

**Figure 2:**
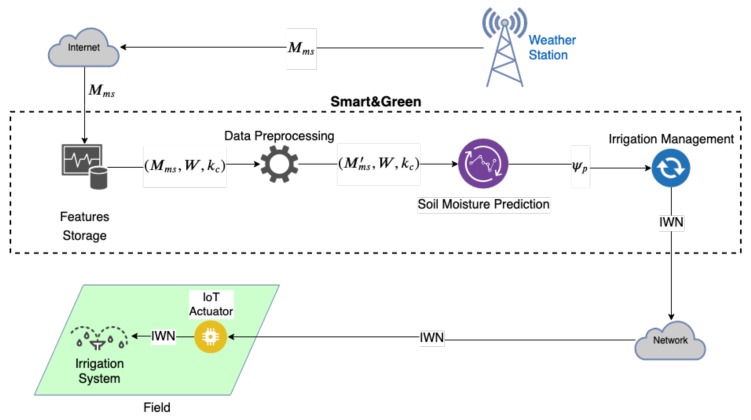
Smart&Green Module for soil moisture prediction.

The Smart&Green framework implements the prediction model as a modular service (Figure 2) to complement the irrigation management of fields without soil moisture sensors (e.g., field 1 in Figure A1). The framework automatically retrieves and stores the meteorological data set $M_{ms}$ published on the Internet by weather stations. We defined $M_{ms}$ in Appendix B.

The framework daily preprocesses the $M_{ms}$ data to remove possible outliers. The module of soil moisture prediction estimates the matric potential $\psi_{p}$ of a field, based on $\left(M_{ms}^{\prime },W,k_{c}\right)$ (Appendix B). The irrigation management module computes the current soil moisture $\theta_{c}$ by applying $\psi_{p}$ on the Van Genutchen [57] model, following Equation (A1). Then, the obtained $\theta_{c}$ is applied to Equation (A2) or (A3) to calculate the irrigation water need (IWN).

In the Internet of Things (IoT) context, the framework sends the IWN value to an actuator to start the irrigation system. The irrigation system stops when the field receives the amount of water specified by the IWN. Although this present work does not handle an automatic irrigation system, this issue is a concern for the full implementation of an IoT system for smart farms.

### 3.2. Service Layer

The Service layer supports the Application layer by storing data provided by Smart&Green users and synchronizing crop, weather station, and soil data when required by any irrigation management module. The Service layer also carries out fusion tasks for data of soil moisture sensors (e.g., fields 2 and 3 of Figure A1) or the soil moisture prediction.

The Storage service provides centralized data persistence for the monitoring of weather, crop, and soil data in the fields. The Synchronization service selects the crop coefficient $k_{c}$ which is appropriate to the current development stage of the crop and retrieves the weather data $M_{ms}$ for the period needed for irrigation management. The service also requests the Communication layer for new data from the Internet, if needed.

In the case of soil data, the Synchronization service selects data from all monitoring points $BT_{c}$ in the field, taking into account the period of irrigation management. If there are no data available, the service requests new data from the user (field 2 of Figure A1) or the Communication layer (Field 3 of Figure A1). Finally, the Synchronization service makes the data set composed of $\left\{k_{c,current},M_{ms,period},BT_{c,period}\right\}$ available.

The Irrigation Management Modules use the Fusion service if there is a field with monitoring points, each with one or more soil moisture sensors (e.g., tensiometers) at different depths *z* (e.g., fields 2 and 3 of Figure A1). The Fusion service can also treat data created in soil moisture prediction when irrigation management is required for fields without soil moisture sensors. Data fusion consists of processing the data to detect and remove outliers (DRO) and submitting data to a co-operative function (CF), according to a multilevel data fusion architecture [58].

DRO tasks apply criteria and algorithms defined by specialist users to immediately detect and remove outliers. The Register module of the Application layer allows for the creation of threshold values for soil data. DRO algorithms are specific to the soil layer monitored. According to [58], the Z-score is more efficient for data series at the most superficial soil layer (e.g., z=15 cm). In contrast, the Generalized ESD (Extreme Studentized Deviate) algorithm works well with data at depth of z=45 cm. The Smart&Green framework has implementations of the Chauvenet, Z-Score, Modified Z-Score, and Generalized ESD algorithms.

A co-operative function (CF) [59] aggregates one type of data gathered at different monitoring points of a field. Smart&Green uses the mean function as the CF to aggregate soil moisture data at the same depth. For example, Field 2 of Figure A1 has six monitoring points at two depths. Therefore, the framework calculates the mean of each set of six samples gathered at a given depth *z*. After fusion, the data are ready for irrigation management at the Application layer, which decides whether irrigation should be started.

### 3.3. Communication Layer

**Figure 3:**
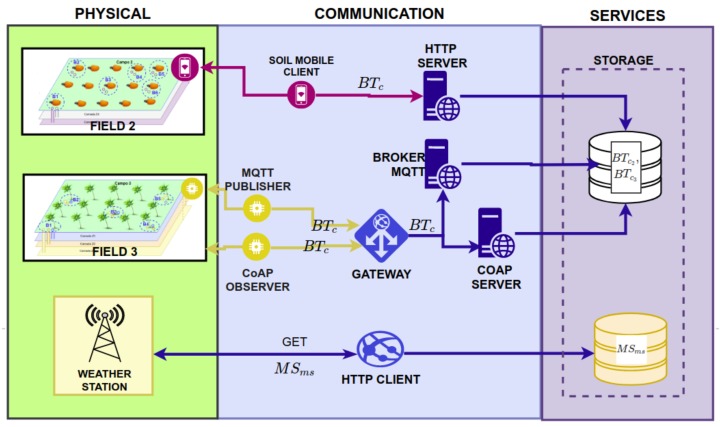
Interaction between the Communication Layer and the Physical and Services Layers.

The Communication Layer has a set of software to gather weather and soil data through network protocols. This includes a weather client, four soil clients, three soil servers, and a gateway (see Figure 3). The weather client consists of an HTTP client which downloads weather station data from the Internet and stores them. Smart&Green offers an API to gather data from Brazilian automatic weather stations [5].

The soil client gathers the matric potential data $\psi_{m}$ if the field has tensiometers installed. If the tensiometers are analog, users have to manually collect the $\psi_{m}$ data, using a mobile application that sends it to the framework via HTTP. If the tensiometers are digital, they are integrated into sensor nodes that send the data to a gateway near the field. The gateway hosts CoAP and MQTT clients, which forward the $\psi_{m}$ data to the servers of the framework.

## 4. Materials and Method

In this section, we list the principal software components used in the implementation of our proposed framework (Section 4.1). We describe what we use for soil moisture prediction, in terms of (1) the weather, crop, and soil moisture information; (2) the criteria for outlier detection and removal; and (3) the tested machine learning techniques (see Section 4.2).

### 4.1. Smart & Green Framework Implementation

We implemented the Smart&Green framework using the Python 3 Language. In the Application layer (Section 3.1), irrigation management by the water balance module uses PyETo [60] to calculate the reference evapotranspiration for a crop, with reference to the Penman-Monteith Model [61]. The crop evapotranspiration measures the water consumption by crops, according to the developmental stage of the crop and weather conditions [3]. The Service Layer uses the MySQL database [62] to support the Storage service. The PyAstronomy library [63] provides the implementation of the Generalized ESD (Extreme Studentized Deviate) algorithm for the Fusion Service.

The aiocoap package [64] supports the implementation of the CoAP client and server of the Communication layer (Section 3.3). The MQTT client uses the Eclipse Paho MQTT client library [65], and we used Mosquito as the MQTT server [66]. Our API for the acquisition of weather data operates by scraping the web page of the automatic Brazilian weather stations [5] using the libraries requests [67] and beautifulsoup4 [68], as well the frameworks Django [69] and Django rest [70]. We implemented the mobile application for soil moisture gathering from fields with analog tensiometers (e.g., field 2 of Figure A1) in the Javascript language through the Firebase platform [71], React Native framework [72], Realm [73], and React Native Firebase [74] libraries. We also provide a web interface for the Smart&Green Framework developed using Django [69]. Smart&Green was run on a machine with 2 GB of RAM, 20 GB of hard disk storage, and the Ubuntu 18.04LTS OS.

For farm fields with sensor nodes, such as field 3 of Figure A1, we used an Arduino Pro Mini and three Irrometer Watermark 200SS [75] digital tensiometers to implement a sensor node which gathers soil moisture data at 15, 45, and 75 cm. The watermark measures the soil water tension (i.e., matric potential), which ranges from 0 to 200 CBar/KPa. The sensor nodes use four AA alkaline batteries of 1.5 v as the power source. We modify the Arduino removing the voltage controller and LEDs to the node has a longer operating life. Sensor nodes send soil data through an nRF24L01 with an external anthem and power amplifier. The nRF24L01 is a single-chip radio transceiver for the 2.4–2.5 GHz ISM band. After data transmission, the sensor node hibernates for an hour to save power [58]. We used a Raspberry Pi 3 with the Raspbian OS as the gateway to receive soil moisture data and send them by the CoAP or MQTT client to storage.

### 4.2. Soil Moisture Prediction

#### 4.2.1. Raw Data Set

**Figure 4:**
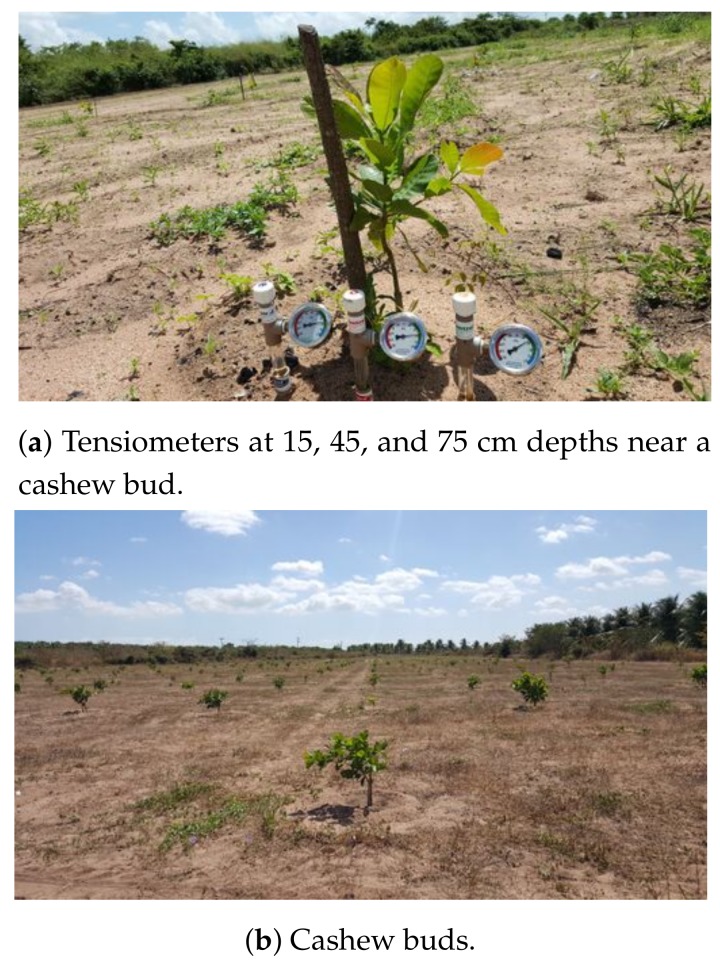
Details of the Experimental Field.

We obtained a data set of soil moisture from an experimental cashew field with other tropical raw materials in the city of Paraibapa, Brazil [76]. The field had approximately 250 cashew trees with nine monitoring spots. Each spot had three tensiometers that allowed manual reading of the matric potential at depths of 15, 45, and 75 cm. Figure 4 shows the details of the analog tensiometers (soil moisture sensors), which provided the data set from 2016, 2017, and 2018, with 234, 245, and 138 daily samples, respectively. It is worth mentioning that, in 2016, the field had salvation irrigation in which we gave 5 liters of water for each bud whenever the installed tensiometer at a depth of 15 cm read a matric potential of 60 kPa. Salvation irrigation prevents plants from dying.

The meteorological data set was obtained from a Brazilian national weather station at Itapipoca city, Ceara (altitude 102 m; latitude $03^{\circ }29^{\prime }$ S; longitude $39^{\circ }35^{\prime }$ W). However, we decide to use the pluviometer sensor data of the field, as the rainfall sensor of the weather station had missing values over an extended period. Table 1 presents the acronyms, the description of the variables, and the measurement data units used in our prediction problem. We release the aggregated data set to the scientific community to ensure the reproducibility of our results and promote research developments in this field (see Supplementary Materials).

We apply the matric potential $\psi_{m}$ (kPa), and the constants in Table 2 to Van Genutchen Model (Equation (A1)) give the soil moisture θ in cm${}^{3}$ of water/cm${}^{3}$ of soil. We obtained in the laboratory the constants which describe the soil type of the experimental field. The irrigation managements use θ to estimate the irrigation water need (Equations (A2) and (A3)).

We also analyzed the soil particle composition of the experimental field. The soil has a medium sandy texture in the layer from 0 to 30 cm (805 g kg${}^{-1}$ of sand, 76 g kg${}^{-1}$ of clay and 119 g kg${}^{-1}$ of silt) and the medium texture in the other layers, presenting the characteristics of a red-yellow Argisol [77]. The maximum organic matter content was 6.4 g dm${}^{-3}$; the pH varied from 5.6 to 6.3 between the layers; the capacity of cations exchange, from 31.3 to 64.8, and the sum of maximum bases was 62% in the most superficial layer.

#### 4.2.2. Outlier Detection and Removal

First, the instances that presented $T_{max},T_{min}<20$, $T_{max},T_{min}>39$, $RH_{max},RH_{min}<20$, and Ri>250 were removed, as these feature ranges are uncommon in the city of Paraibapa. After that, the mean μ and the standard deviation σ were computed for the variables *P*, Rn, and $U_{2}$ for each month. Afterward, we filtered out data instances with anomalies; that is, the instances with distance to μ greater or equal than 3×σ were removed, as performed in [78].

#### 4.2.3. Machine Learning Methods

We decided to build the prediction model using algorithms which have been widely applied to solve regression problems: Linear Regression [46], Decision Stump [47], M5 Model Trees and Rules (M5P) [48,49], Random Tree, Random Forest [50], and RepTree. Weka [79] provided all of these. Another widely used algorithm is the Gradient Boosting Regression Tree (GBRT) [51,52] method and its implementation XGBoost [80].

Linear regression is a linear model of the relationship between a scalar response (in this work, the matric potential $\psi_{m}$) and one or more explanatory variables (see Table 1). The linear model consists of linear predictor functions which use the data to estimate the unknown parameters [46]. Decision stump models a one-level decision tree. The tree has one root (an internal node) which connects the leaves (terminal nodes). The model uses the value of one single feature to make predictions [47].

M5P produces a tree with a multilinear regression model for each node. A greedy algorithm removes the insignificant features in the model fitted to each node. Then, the terminal node computes new predicted values, considering the predicted values of other intermediate nodes between the terminal node and the root node [48,49].

Random Tree (RT) uses if-then conditions for answering questions in a sequential order to achieve a specific result. The depth of the tree consists of the number of questions needed to reach a prediction value. Random Forest (RF) is a collection of decision trees that can use a random subset of the features. Therefore, the number of trees impacts the number of features used and limits the number of errors due to bias or variance [50].

RepTree is a fast decision tree algorithm in Weka which uses information gain/variance to build a regression tree. Reduced-error pruning takes into account the method backfitting [79]. GBRT is a predictive model based on a weak learner, a loss function, and an additive model. The weak learner is a decision tree that can be modified to achieve better results. The additive model adds weak learners to correct for the residual errors of all previous trees. The loss function consists of the mean squared error for regression problems [51,52].

We set some hyperparameters required by RT, RF, RepTree, and GBRT with the default values of Weka [79] and XGboost [80]. We set the maximum tree depth (max_depth) to 3 for GBRT and to without restriction for the RT, RF, and RepTree methods. The number of trees (n_estimators) was 100 for all methods. Finally, GBRT required a third hyperparameter, the learning rate (learning_rate), which we set to 0.1.

The training and test sets included, respectively, 80% and 20% of the data from each soil moisture sensor at a depth of 15 cm. Overall, for each possible combination, we generated a model and picked the model which gave the lowest MAE (Mean Absolute Error) and RMSE (Root Mean Square Error)—metrics which have been widely used to measure the quality of a prediction model (see Equations (A6) and (A7) in Appendix B).

## 5. Results

First, we employed the outlier removal techniques of Fusion Service (Section 3.2) on the real soil moisture data in order to analyze the influence of these techniques on irrigation management (Section 3.1.1) in the Smart&Green framework (Section 5.1). Then, we analyzed the MAE and RMSE of the machine learning techniques tested with the local and global approaches (Section 3.1.2) proposed for the Smart&Green soil moisture prediction module (Section 5.2). Finally, we applied the soil moisture data created by the best prediction models for irrigation management to analyze the water savings (Section 5.3).

### 5.1. Impact of Outlier Removal Techniques on Real Moisture Data by Irrigation Management

From the raw data set (Section 4.2), we selected data from from April to October/2017, since there was no salvation irrigation in the experimental field, which could affect the irrigation water need (IWN) estimated by water balance and matric potential management. Then, we set Fusion Service (Section 3.2) to use an outlier removal technique to process the matric potential $\psi_{c}$ provided by the nine tensiometers (soil moisture sensors) at 15 cm of depth in the experimental field (Section 4.2). Second, the Fusion Service aggregated $\psi_{c}$ data using the co-operative function mean. After this, the modules of water balance and potential matric (Section 3.1) applied the aggregated $\psi_{c}$ to Van Genutchen Model (Equation (A1)) to estimate the current soil moisture $\theta_{c}$ at the crop root zone (z= 30 cm) and compute IWN.

Water balance management (Equation (A3)) can depend only on the crop evapotranspiration $ET_{c}$ (Equation (A4)) and rainfall *R* to estimate the IWN of a crop. Therefore, we can only use weather data and the crop coefficient (Table 1) to plan water balance for *n* days. However, the IWN estimation is more precise when we can incorporate the current soil moisture data $\theta_{c}z$ since the water storage in the crop root zone can contribute to the crop water supply. We set water balance to estimates IWN every three days (n=3) over six months (26 samples of *n* intervals), and the results in Table 3 indicate that the use of $\theta_{c}z$ saved an average of 90.4% of the IWN estimated, as compared to that using only weather and crop data.

**Figure 5:**
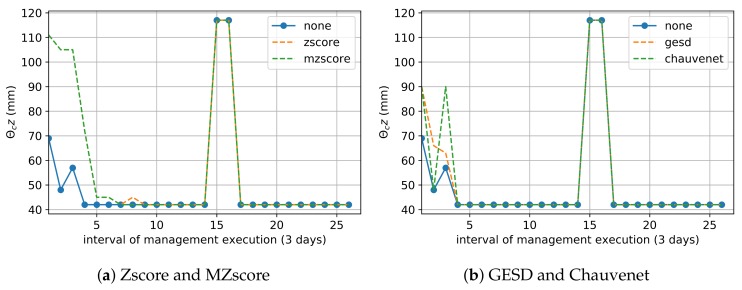
Real Moisture Data used in Water Balance—the points outside of the dashed lines are the current moisture data. $\theta_{c}z$ modified by an outlier removal technique.

The preprocessing of real data with Zscore and MZscore techniques optimized the tradeoff reasonably between IWN saving and healthy crop development, since these outlier removal techniques decreased this result to 89.3%. Zscore and MZscore data treatment led to an increment of the moisture $\theta_{c}$ values (Figure 5a), and, consequently, a reduction of the term ΔA of water balance (Equation (A3)). GESD and Chauvenet did not affect the moisture $\theta_{c}$ data enough (Figure 5b), as well as IWN saving (Table 3).

**Figure 6:**
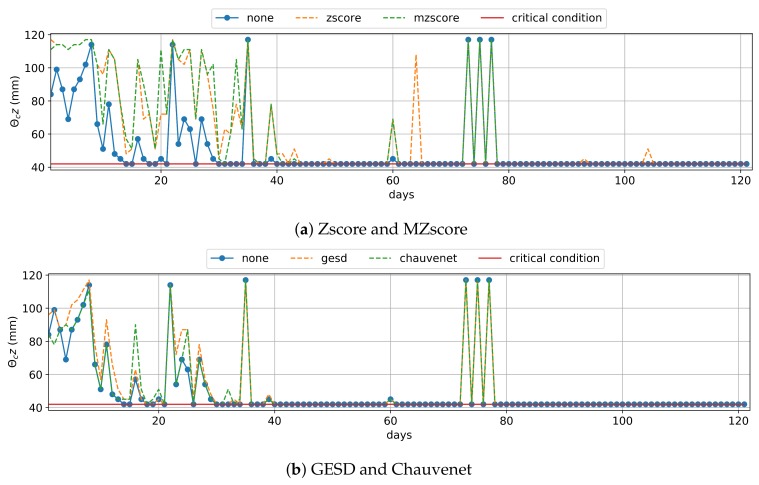
Real Moisture Data used in Matric Potential—the points outside of the dashed lines are soil moisture $\theta_{c}$ modified by an outlier removal technique. Irrigation occurs when $\theta_{c}$ reaches the critical condition $\theta_{cr}$ (red line).

The matric potential approach depends exclusively on the soil moisture data (Equation (A2)), and we planned this irrigation management to estimate the daily IWN over six months (121 samples of days) using the current moisture $\theta_{c}$ data at the shallowest crop root zone (e.g., z=30 cm). In the matric potential approach, irrigation occurs only when $\theta_{c}$ is minor or equal to the critical moisture condition $\theta_{cr}$, which is the ideal moisture for irrigation and signals when the crop productivity is starting to decline. Table 3 presents the IWN saving of matric potential management that used an outlier removal technique when compared to the one that did not use them. Figure 6 shows that there were several days where $\theta_{c}$ was equal to $\theta_{cr}$ (day 1 to 13). However, all outlier remotion techniques treated various outliers that reduced the current moisture (e.g., 14th and 15th; 18th and 19th; 26th days), improving the water savings, on average, by 4.3% to 20.7% (Table 3). Zscore and MZscore (Figure 6a) contributed more to the tradeoff between IWN saving and healthy crop development because they increased the current moisture $\theta_{c}$ above the critical condition $\theta_{cr}$ in more days(e.g., the 40th to 43th; 64th; 104th days) than GESD and Chauvenet (Figure 6b).

### 5.2. Performance of Models for Soil Moisture Prediction

The main goal of this analysis is to evaluate and establish the best machine learning (ML) technique among those tested for each approach (local and global prediction models). In this context, we considered analog soil sensors (tensiometers) distributed throughout a large field (Figure 4). We compared the following ML techniques: Linear Regression, Decision Stump, M5P, Random Tree, Random Forest, RepTree, and Gradient Boosting. Through the metrics MAE (Mean Absolute Error) and RMSE (Root Mean Square Error), we evaluated the local and global approaches for the estimation of the matric potential $\psi_{m}$—the primary variable for soil moisture prediction (Appendix B). Finally, we used the 99% confidence intervals to determine the best-performing model.

Table 4 presents the MAE and RMSE retrieved by the local approach for each prediction algorithm. The results of the local approach are the mean of the MAE and RMSE achieved by the nine models built for each monitoring point of the experimental field. Considering all the analyzed errors, Gradient Boosting outperformed all the other techniques, followed by Random Forest. Both approaches presented acceptable errors, according to a specialist agronomic engineer.

Table 5 presents the MAE and RMSE retrieved by the global approach for each algorithm. Again, the Gradient Boosting algorithm achieved the best results, compared to all other evaluated models. It can be noticed that the MAE and RSME of the Gradient Boosting model did not vary as much as the other evaluated models; this means the global model generalizes well for any data from any sensor in the field. The Local Gradient Boosting approach outperformed the global one. However, even though the global model error had an average MAE error increase of 58.9% and average RMSE error increase of 45.7%, the errors were still acceptable, according to the specialist agronomic engineer.

**Figure 7:**
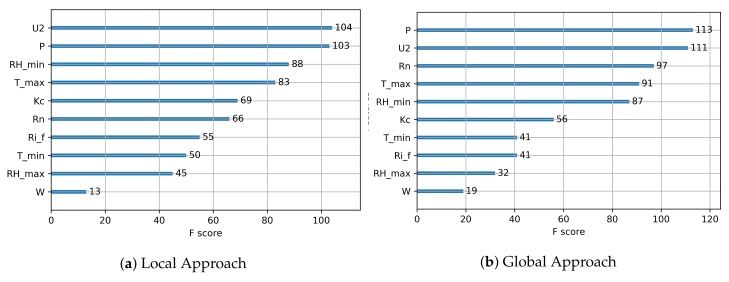
Analysis of the relevance of features by gradient boosting prediction approach.

We also analyzed the relevance of the features of Table 1, in the context of the local and global approaches. We employed Gradient Boosting as, according to the results of this section, it has proved to be the best ML technique for our purposes. XGboost [80] estimates the relevance of each feature by counting the number of times it is used in a split node of any decision tree in the Gradient Boosting forest. Figure 7 presents the results. The more an attribute is used in a decision tree, the higher its relative importance is. The most-used variables in both approaches were $U_{2}$, *P*, $T_{max}$, $RH_{min}$, Rn, and $k_{c}$.

### 5.3. Analysis of Water Savings through the Use of Predicted Data of Soil Moisture

We used the local and global models based on Gradient Boosting to predict the matric potential $\psi_{m}$ for a field without soil moisture sensors over six months. The local approach estimated daily $\psi_{p}$ at a depth of 15 cm, as the field had nine monitoring points. Therefore, we could apply the outlier removal techniques to treat this data set of nine daily values. The global approach estimated one daily value for the $\psi_{p}$ at a depth of 15 cm in the field. In this case, there was no need for preprocessing the data using the outlier removal techniques. We use $\psi_{p}$ to compute predicted soil moisture data for the crop root zone (z=30 cm) through the Van Genutchen Model (Equation (A1)).

For water balance (Equation (A3)), executed every three days (n=3) for six months (26 samples of *n*), we compared the benefits of our computational models by analyzing how much they saved, in terms of the irrigation water need (IWN) estimated using only weather and crop data. Table 6 shows that the use of global approach data saved an average of 56.4% and that the local approach without data preprocessing saved 100% of the IWN on average.

**Figure 8:**
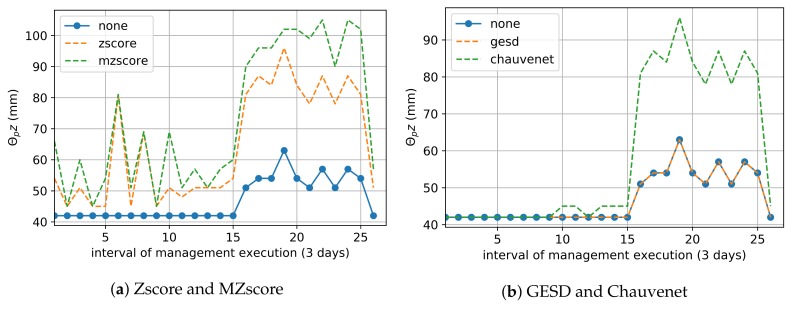
Predicted Moisture Data used inWater Balance—the points outside of the dashed lines are soil moisture data $\theta_{c}$ modified by an outlier removal technique.

The Zscore and MZscore methods tended to treat outliers by removing values that decreased the current moisture $\theta_{c}$ (Figure 8a). Therefore, they reduced the result of the local predicted data by 90% and 75.6%, respectively, for water balance. The employment of Chauvenet for the local approach data also saved 90% of IWN on average since it almost achieved the performance of Zscore (Figure 8b). The use of GESD did not affect the local approach predicted data (Figure 8b), such that it had almost the same water savings as when no outlier removal technique was employed (Table 6). We consider the global approach and local approach using Zscore, MZscore and Chauvenet techniques optimized the tradeoff between IWN saving and healthy crop development, and they did not outweigh the IWN savings of water balance that used real soil moisture $\theta_{c}$ (Table 3).

**Figure 9:**
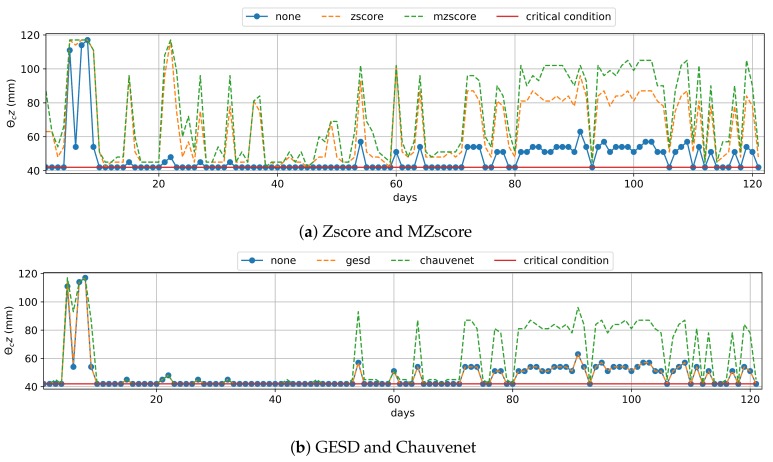
Predicted Moisture Data used in Matric Potential—the points outside of the dashed lines are current soil moisture $\theta_{c}$ data modified by an outlier removal technique. Irrigation occurs when $\theta_{c}$ is minor or equal to the critical moisture condition $\theta_{cr}$ (red line).

For the matric potential (Equation (A2)) executed daily, irrigation occurs when the current moisture $\theta_{c}$ is minor or equal to the critical moisture condition $\theta_{cr}$, which is the ideal moisture for irrigation, signaling when the crop productivity is starting to decline. To compare the results of using the predicted data we used, as a baseline, the IWN estimated by the matric potential using real moisture data without an outlier removal technique. The use of the global approach data saved an average of 53.1% when compared to the IWN baseline, and the use of local approach data without preprocessing saved 100% (Table 6). The Zscore, MZscore, and Chauvenet techniques tended to remove the outliers that decreased the moisture values, setting the current moisture higher than the critical condition (Figure 9). GESD kept the performance of matric potential management that used the local approach data. However, we do not recommend the use of predicted data with the matric potential management, as the rise of IWN saving outweighed the results related to the use of real moisture data in this same irrigation management example (Table 3).

## 6. Conclusions

Smart Agriculture presently lacks an Internet of Things (IoT) platform for the gathering, preprocessing, and storage of data used in irrigation management, which also allows for the reuse of code to different sets of crops, soil, irrigation system, and weather data sources. In this paper, we have proposed the Smart&Green IoT framework which executes an irrigation management plan using the water balance and potential matric approaches according to the crop, as well as irrigation system information provided by users and the weather and soil moisture data available close to the monitored fields. Smart&Green can be used to preprocess soil moisture data with outlier removal criteria and techniques of Zscore, MZscore, GESD, and Chauvenet to provide a more precise irrigation water need (IWN) in irrigation management.

For large fields without soil moisture sensors, we have considered the problem of predicting the soil moisture by analyzing the weather data, crop coefficients, and amount of irrigation water. We have formally introduced the problem of soil prediction and designed a methodology which uses training models according to two different approaches: Local and global. These approaches determine the implementation of prediction models based on supervised machine learning techniques.

The local approach trains a model for each soil sensor in the field, achieving high performance. However, this approach can lead to a high cost of data management if there are a large number of sensors, as it requires the training and maintainence of a large number of distinctive prediction models. On the other hand, the global approach trains a single prediction model over the observations of all the sensors. Our results show that gradient boosting with regression trees (GBRT) outperformed the other evaluated techniques in both approaches. The local approach had the best results with gradient boost, although the results using the global prediction model were also acceptable.

Furthermore, we have designed a module to support irrigation management with our prediction models, based on GBRT. We evaluated irrigation management using the water balance and matric potential approaches with real and predicted data of soil moisture, as well as the impact of preprocessing data with outlier removal techniques. The real data was part of a data set collected from an experimental field for research in the city of Paraipaba, Brazil, in which cashews and other raw materials were grown. We also report that the dataset will be made publicly available to ensure the reproducibility of our results and promote research developments in this field (see Supplementary Materials).

The use of real soil moisture data from the data set in the water balance approach saved as much as 90.4% of the IWN obtained for the water balance when using only weather and crop data. Zscore and MZscore applied to the real moisture data saved 89.3% of IWN, optimizing the tradeoff between IWN saving and healthy crop development. For the matric potential approach, Zscore and MZscore obtained (on average) 20.7% and 14.1% of the IWN savings obtained by matric potential without the use of any outlier removal technique. Considerating a field without soil moisture sensors, we obtained a reduction between 56.4% and 90% of the water balance IWN based on only weather and crop data, when we use our global and local approaches to predict the soil moisture data. Zscore, MZscore, and Chauvenet preprocess the predicted data to optimize the tradeoff without outweighing the performance of water balance using real moisture data. We do not recommend the use of predicted data in the matric potential approach, as the computational models (with errors) outperform mostly the same approach using real data in terms of water savings, according to our results.

## Figures and Tables

**Table 1:** Raw dataset structure.

Acronym	Description	Unit
Tx–y	Tensiomenter reading at *y* depth in a point of monitoring *x*	kPa
Wx	The water amount given to the crop field in a point of monitoring *x* through irrigation	L
T_max	maximum temperature of air	${}^{\circ }C$
T_min	minimum temperature of air	${}^{\circ }C$
RH_max	maximum relative humidity	%
RH_min	minimum relative humidity	%
Rn	net radiation	MJm${}^{2}$d${}^{-1}$
U2	wind speed	m/s
P	atmospheric pressure	kPa
Ri_f	rainfall gathered by the pluviometer sensor	mm
Kc	crop coefficient	

**Table 2:** Van Genutchen Constants for the Experimental Field.

$\theta_{r}$	$\theta_{s}$	α	*n*
0.14010	0.38839	0.022504	20.524

**Table 3:** Percentage of water saved over six months by irrigation management using real soil moisture data.

Irrigation Management	Outlier Removal Technique	Mean (%)	Confidence Interval (90%)
Water Balance	None	90.4	(81.6, 99.1)
Water Balance	Zscore	89.3	(80.4, 98.3)
Water Balance	MZscore	89.3	(80.4, 98.3)
Water Balance	GESD	90.4	(81.6, 99.1)
Water Balance	Chauvenet	90.4	(81.6, 99.1)
Matric Potential	Zscore	20.7	(13.7, 27.6)
Matric Potential	MZscore	14.1	(8.1, 20.1)
Matric Potential	GESD	4.3	(0.8, 7.9)
Matric Potential	Chauvenet	5.4	(1.5, 9.3)

**Table 4:** Evaluation of ML techniques using the Local Approach: Mean of MAE and RMSE, 99% confidence interval. The best performers are highlighted in **bold**.

Algorithm	MAE	Conf. Interval MAE	RMSE	Conf. Interval RMSE
Linear Regression	0.1408	(0.1318, 0.1498)	0.1730	(0.1642, 0.1818)
Decision Stump	0.1798	(0.1632, 0.1965)	0.2196	(0.2031, 0.2360)
M5P	0.1288	(0.1159, 0.1416)	0.1722	(0.1576, 0.1868)
Random Tree	0.1443	(0.1319, 0.1567)	0.2120	(0.1877, 0.2363)
Random Forest	0.1189	(0.1025, 0.1352)	0.1551	(0.1393, 0.1709)
RepTree	0.1227	(0.1119, 0.1336)	0.1684	(0.1566, 0.1801)
**Gradient Boosting**	**0.0752**	**(0.0683, 0.0822)**	**0.1038**	**(0.0939, 0.1137)**

**Table 5:** Evaluation of ML techniques using the Global Approach: Mean of MAE and RMSE, 99% confidence interval. The best performers are highlighted in **bold**.

Algorithm	MAE	Conf. Interval MAE	RMSE	Conf. Interval RMSE
Linear Regression	0.1628	(0.1510, 0.1746)	0.1993	(0.1881, 0.2110)
Decision Stump	0.1938	(0.1812, 0.2063)	0.2335	(0.2220, 0.2450)
M5P	0.1461	(0.1348, 0.1573)	0.1824	(0.1706, 0.1942)
Random Tree	0.1494	(0.1413, 0.1574)	0.2094	(0.2004, 0.2183)
Random Forest	0.1406	(0.1317, 0.1494)	0.1873	(0.1769, 0.1977)
RepTree	0.1438	(0.1362, 0.1515)	0.1832	(0.1740, 0.1924)
**Gradient Boosting**	**0.1382**	**(0.1382, 0.1382)**	**0.1717**	**(0.1717, 0.1717)**

**Table 6:** Percentage of water saved in six months by irrigation management using predicted soil moisture data.

Irrigation	Prediction	Outlier Removal	Mean	Confidence Interval
Management	Approach	Technique	(%)	(90%)
Water Balance	Global	None	56.4	(41.4, 71.5)
Water Balance	Local	None	100	(100, 100)
Water Balance	Local	Zscore	90.0	(85.7, 94.3)
Water Balance	Local	MZscore	75.6	(67.1, 84.2)
Water Balance	Local	GESD	100	(100, 100)
Water Balance	Local	Chauvenet	90.0	(85.7, 94.3)
Matric Potential	Global	None	53.1	(44.5, 61.7)
Matric Potential	Local	None	43.3	(34.7, 51.9)
Matric Potential	Local	Zscore	95.6	(92.1, 99.2)
Matric Potential	Local	MZscore	97.8	(95.3, 100)
Matric Potential	Local	GESD	43.3	(34.7, 51.9)
Matric Potential	Local	Chauvenet	62.9	(54.6, 71.3)

## Supplementary Materials

The raw data set is available at: http://smartgreen.great.ufc.br/publications.

## Appendix A. Irrigation Management

Figure A1 represents some examples of configurations composed of a crop, soil, weather, and an irrigation system. The house is next to the entrance of the farm and has a connection to the Internet. The house also hosts devices that connect sensor nodes in the field to the Internet. Automatic weather stations make weather data available on the Internet.

In this work, we address fields with one type of crop whose soil has analog (Field 2) or digital tensiometers (Field 3) monitoring the moisture at more than one depth *z*. Tensiometers installed at different points *b* of the field gather readings of the matric potential $\psi_{m}$. Mathematical models use $\psi_{m}$ to measure the soil moisture θ as the level of water retention in the type of soil. We use the Van Genuchten Model [57] to express θ as the volume of water by the volume of soil (cm${}^{3}$/cm${}^{3}$), as Equation (A1) shows:

(A1) $$\theta \left(\psi_{m}\right)=\theta_{r}+\frac{\theta_{s}-\theta_{r}}{\left[1+(\alpha \times \right|\psi_{m}{\left|\right)}^{n}{]}^{1-\frac{1}{n}}},$$

where $\theta_{r}$ is the residual water content, $\theta_{s}$ is the saturated water content, α is related to the inverse of the air entry suction, and *n* is a measure of the pore-size distribution in the soil (e.g., Table 2 has these Van Genutchen constants for the experimental field soil).

The management of matric potential estimates the irrigation water need (IWN) for a field with soil sensors (e.g., Field 3 of Figure A1) taking into account the soil moisture at the monitored depths $z_{i}$, as Equation (A2) shows:

(A2) $$IWN=\frac{\left(\theta_{fc,z_{1}}-\theta_{cr,z_{1}}\right)\times z_{1}+\left(\theta_{fc,z_{2}}-\theta_{c,z_{2}}\right)\times z_{2}+\left(\theta_{fc,z_{3}}-\theta_{c,z_{3}}\right)\times z_{3}}{Ef},$$

where:

- $z_{i}$ represent the thickness of soil along the profile (monitored depth);
- $\theta_{fc}$ represents the field capacity at a depth *z* after the drainage of water excess, which is a constant obtained by a laboratory soil analysis;
- $\theta_{cr}$ is the ideal moisture for irrigation, signaling when the crop productivity starts to decline;
- $\theta_{c}$ is the current soil moisture. In matric potential management, we initialize the irrigation by $\theta_{c,z_{1}}\leq \theta_{cr}$; and
- Ef is the efficiency of the irrigation system used in the field.

Water balance management computes the IWN for an interval of *n* days, comparing the inputs and outputs of water in the soil by using the daily crop evapotranspiration $ET_{c}$, the rainfall *R*, and $\Delta A=(\theta_{fc}-\theta_{c})z$ which represents the soil moisture at the crop root zone *z* through Equation (A3).

(A3) $$IWN=\frac{\sum_{i=1}^{n}\left(ET_{c,i}-R_{i}\right)-\Delta A}{Ef}.$$

The value Ef is the efficiency of the irrigation system placed in the field. The crop evapotranspiration $ET_{c}$ represents the water consumption by a type of crop at its current life development stage, which is given by:

(A4) $$ET_{c}=ET_{o}\times k_{c}.$$

The Penman-Monteith model [61] estimates the reference evapotranspiration $ET_{o}$ using weather data. Each type of crop, without hydrological restriction, has a specific coefficient curve in which the crop coefficient $k_{c}$ varies from 0 to 1.5 with the number days since the planting of the crop.

## Appendix B. The Problem of Soil Moisture Prediction

Let $B=\{b_{1},\cdots ,b_{n}\}$ be the set of tensiometers monitoring the soil moisture of a field at *n* different points. A tensiometer $b_{j}$ gathers the sample $\left(\psi_{j,15},\psi_{j,45},\psi_{j,75}\right)$, which consists of the matric potential ψ readings at 15, 45, and 75 cm depths, respectively, at a point *j*. Let $W=\{w_{1},\cdots ,w_{n}\}$ be the set of water amount given to the crop at *n* different points by an irrigation system. Irrigation management calculates *W* using the Equations (A2) and (A3), for example.

The weather data are required, as the climatic conditions affect a crop’s need for soil water and rainfall can increase the soil moisture. Therefore, we assume that there is a weather station ms close to the field which provides the meteorological data set $M_{ms}=\left\{T_{min},T_{max},RH_{min},RH_{max},Rn,U_{2},P,R_{i}\right\}$, where:

- $T_{min}$ and $T_{max}$ are the minimum and maximum air temperature, respectively;
- $RH_{min}$ and $RH_{max}$ are the minimum and maximum relative humidity, respectively;
- $R_{n}$ is the net radiation;
- $U_{2}$ is the wind speed;
- *P* is the atmospheric pressure; and
- $R_{i}$ is the rainfall.

Within a daily time interval, there are several observations about the weather and field. We denote by *O* the collection of observations, which consists of (*t*, *B*, $M_{ms},W,k_{c}$), recorded within a time *t* over a day. The irrigation scheduling methods presented in Appendix A decide to start precise irrigation when the current moisture $\theta_{c,z_{1}}$ is low at the superficial layer of soil ($z_{1}=15$ cm). Therefore, the matric potential $\psi_{i,15}$ is analyzed daily to achieve $\theta_{c,z_{1}}$ using the Van Genutchen model (Equation (A1)). In this work, we wish to monitor the level of soil moisture without soil sensors (i.e., tensiometers).

Let $y(i,M_{ms},w_{i},k_{c})$ be a function that, given the monitoring point *i*, the meteorological variables $M_{ms}$, the irrigation water amount $w_{i}$ at the point *i*, and the crop coefficient $k_{c}$ returns $\psi_{i,15}$ (the matric potential at a depth of 15 cm). Then, $y\left(i,M_{ms},w_{i},k_{c}\right)=\psi_{i,15}$. We define the problem of soil moisture prediction as the problem of finding an accurate function *f* for predicting $y(i,M_{ms},w_{i},k_{c})$, given all the observations recorded in *O*.

**Definition** **A1.** *The problem of soil moisture prediction requires finding a predictive function*$\hat{f}$*over the class of all possible predictive functions H, such that*

(A5) $$\hat{f}=argmin\Delta \left(f\right),$$

*where*f∈H*and* Δ *is a loss function assessing the quality of a candidate predictive function f over the observations in O. In this work, we use the Root Mean Squared Error (RMSE) and the Mean Absolute Error (MAE) loss functions, defined as:*

(A6) $$\Delta_{RMSE}\left(f\right)=\sqrt{\frac{1}{|O^{\prime }|}\sum_{\left(i,M_{ms},w_{i},k_{c}\right)\in O^{\prime }}{\left(f\left(i,M_{ms},w_{i},k_{c}\right)-y\left(i,M_{ms},w_{i},k_{c}\right)\right)}^{2}},\;and$$

(A7) $$\Delta_{MAE}\left(f\right)=\frac{1}{|O^{\prime }|}\sum_{\left(i,M_{ms},w_{i},k_{c}\right)\in O^{\prime }}\left|f\left(i,M_{ms},w_{i},k_{c}\right)-y\left(i,M_{ms},w_{i},k_{c}\right)\right|,$$

*where O’ is the set of observations used to assess the quality of the prediction and* $f(i,M_{ms},w_{i},k_{c})$ *is the estimate returned by function f for* $y(i,M_{ms},w_{i},k_{c})$*. The smaller the value yielded by the above loss functions, the better the predictive performance of f.*

We employ Machine Learning (ML) techniques to address the problem of soil moisture prediction. More specifically, we aim at learning, from the observations in *O*, some function $\hat{f}$ that minimizes the error measured by Δ. We train the prediction models on a data set containing examples built from past sensor observations (see Section 4.2). The ML techniques for regression tasks used in this work are: Linear Regression [46], Decision Stump [47], M5 Model Trees and Rules [48,49], Random Tree, Random Forest [50], RepTree, and Gradient Boosting Regression Tree [51,52].

## References

[1] FAO (2014). World Agriculture: Towards 2015/2030—An FAO Perspective.

[2] Haverkort A. (2006). Handbook of Precision Agriculture. Principles and Applications. Euphytica.

[3] ANA (2017). Atlas Irrigação: Uso Da água Na Agricultura Irrigada.

[4] Voutos Y., Mylonas P., Katheniotis J., Sofou A. (2019). A Survey on Intelligent Agricultural Information Handling Methodologies. Sustainability.

[5] INMET Brazilian Automatic Weather Station of INMET (Instituto Nacional de Meteorologia). http://www.inmet.gov.br/portal/index.php?r=estacoes/estacoesautomaticas.

[6] Borgia E. (2014). The Internet of Things vision: Key features, applications and open issues. Comput. Commun..

[7] Alam F., Mehmood R., Katib I., Albogami N.N., Albeshri A. (2017). Data Fusion and IoT for Smart Ubiquitous Environments: A Survey. IEEE Access.

[8] Pang Z., Chen Q., Han W., Zheng L. (2015). Value-centric Design of the Internet-of-things Solution for Food Supply Chain: Value Creation, Sensor Portfolio and Information Fusion. Inf. Syst. Front..

[9] Talavera J.M., Tobón L.E., Gómez J.A., Culman M.A., Aranda J.M., Parra D.T., Quiroz L.A., Hoyos A., Garreta L.E. (2017). Review of IoT applications in agro-industrial and environmental fields. Comput. Electron. Agric..

[10] Abaya S., De Vega L., Garcia J., Maniaul M., Redondo C.A. A self-activating irrigation technology designed for a smart and futuristic farming. Proceedings of the 2017 International Conference on Circuits, Devices and Systems (ICCDS).

[11] Math R.K., Dharwadkar N.V. A wireless sensor network based low cost and energy efficient frame work for precision agriculture. Proceedings of the 2017 International Conference on Nascent Technologies in Engineering (ICNTE).

[12] Rajkumar M.N., Abinaya S., Kumar V.V. Intelligent irrigation system—An IOT based approach. Proceedings of the 2017 International Conference on Innovations in Green Energy and Healthcare Technologies (IGEHT).

[13] Santoshkumar, Udaykumar R.Y. Development of WSN system for precision agriculture. Proceedings of the 2015 International Conference on Innovations in Information, Embedded and Communication Systems (ICIIECS).

[14] Mesas-Carrascosa F., Santano D.V., Meroño J., de la Orden M.S., García-Ferrer A. (2015). Open source hardware to monitor environmental parameters in precision agriculture. Biosyst. Eng..

[15] Balamurugan C., Satheesh R. (2017). Development of Raspberry pi and IoT Based Monitoring and Controlling Devices for Agriculture. J. Soc. Technol. Environ. Sci..

[16] Flores K.O., Butaslac I.M., Gonzales J.E.M., Dumlao S.M.G., Reyes R.S.J. Precision agriculture monitoring system using wireless sensor network and Raspberry Pi local server. Proceedings of the 2016 IEEE Region 10 Conference (TENCON).

[17] Maia R.F., Netto I., Tran A.L.H. Precision agriculture using remote monitoring systems in Brazil. Proceedings of the 2017 IEEE Global Humanitarian Technology Conference (GHTC).

[18] Heble S., Kumar A., Prasad K.V.V.D., Samirana S., Rajalakshmi P., Desai U.B. A low power IoT network for smart agriculture. Proceedings of the 2018 IEEE 4th World Forum on Internet of Things (WF-IoT).

[19] Sathish kannan K., Thilagavathi G. Online farming based on embedded systems and wireless sensor networks. Proceedings of the 2013 International Conference on Computation of Power, Energy, Information and Communication (ICCPEIC).

[20] Kamelia L., Ramdhani M.A., Faroqi A., Rifadiapriyana V. (2018). Implementation of Automation System for Humidity Monitoring and Irrigation System. IOP Conf. Ser. Mater. Sci. Eng..

[21] Navarro-Hellín H., Torres-Sánchez R., Soto-Valles F., Albaladejo-Pérez C., López-Riquelme J., Domingo-Miguel R. (2015). A wireless sensors architecture for efficient irrigation water management. Agric. Water Manag..

[22] Shelby Z., Hartke K., Bormann C. (2014). The Constrained Application Protocol (CoAP). https://rfc-editor.org/rfc/rfc7252.txt.

[23] (2014). OASIS Message Queuing Telemetry Transport (MQTT). http://docs.oasis-open.org/mqtt/mqtt/v3.1.1/os/mqtt-v3.1.1-os.html.

[24] Byishimo A., Garba A. (2016). Designing a Farmer Interface for Smart Irrigation in Developing Countries.

[25] Popovic T., Latinović N., Pesic A., Zecevic Z., Krstajic B., Đukanović S. (2017). Architecting an IoT-enabled platform for precision agriculture and ecological monitoring: A case study. Comput. Electron. Agric..

[26] Dinh Le T., Tan D.H. Design and deploy a wireless sensor network for precision agriculture. Proceedings of the 2015 2nd National Foundation for Science and Technology Development Conference on Information and Computer Science (NICS).

[27] Hamouda Y., Msallam M. (2018). Smart heterogeneous precision agriculture using wireless sensor network based on extended Kalman filter. Neural Comput. Appl..

[28] Figueroa M., Pope C. (2017). Root System Water Consumption Pattern Identification on Time Series Data. Sensors.

[29] Ferrandez J., Manuel García-Chamizo J., Nieto-Hidalgo M., Mora-Martínez J. (2018). Precision Agriculture Design Method Using a Distributed Computing Architecture on Internet of Things Context. Sensors.

[30] Patokar A., Gohokar V. (2018). Precision Agriculture System Design Using Wireless Sensor Network. Information and Communication Technology.

[31] Vaishali S., Suraj S., Vignesh G., Dhivya S., Udhayakumar S. Mobile integrated smart irrigation management and monitoring system using IOT. Proceedings of the 2017 International Conference on Communication and Signal Processing (ICCSP).

[32] Pavón-Pulido N., López-Riquelme J.A., Torres R., Morais R., Pastor J.A. (2017). New trends in precision agriculture: A novel cloud-based system for enabling data storage and agricultural task planning and automation. Precis. Agric..

[33] Karimi N., Arabhosseini A., Karimi M., Kianmehr M. (2018). Web-based monitoring system using Wireless Sensor Networks for traditional vineyards and grape drying buildings. Comput. Electron. Agric..

[34] Mat I., Kassim M.R.M., Harun A.N. Precision agriculture applications using wireless moisture sensor network. Proceedings of the 2015 IEEE 12th Malaysia International Conference on Communications (MICC).

[35] Mat I., Kassim M., Harun I.A.N. Precision Irrigation Performance Measurement Using Wireless Sensor Network. Proceedings of the 2014 Sixth International Conference on Ubiquitous and Future Networks (ICUFN).

[36] Caetano F., Pitarma R., Reis P. (2015). Advanced System for Garden Irrigation Management. Adv. Intell. Syst. Comput..

[37] Balaji Bhanu B., Hussain M.A., Ande P. Monitoring of soil parameters for effective irrigation using Wireless Sensor Networks. Proceedings of the 2014 Sixth International Conference on Advanced Computing (ICoAC).

[38] Capraro F., Tosetti S., Vita Serman F. (2014). Supervisory control and data acquisition software for drip irrigation control in olive orchards: An experience in an arid region of Argentina. Acta Horticult..

[39] Miller L., Vellidis G., Mohawesh O., Coolong T. (2018). Comparing a Smartphone Irrigation Scheduling Application with Water Balance and Soil Moisture-based Irrigation Methods: Part I—Plasticulture-grown Tomato. HortTechnology.

[40] Sawant S., Durbha S., Jagarlapudi A. (2017). Interoperable agro-meteorological observation and analysis platform for precision agriculture: A case study in citrus crop water requirement estimation. Comput. Electron. Agric..

[41] Mauget S., Leiker G. (2010). The Ogallala Agro-Climate Tool. Comput. Electron. Agric..

[42] Carlesso R., Petry M., Trois C. The Use of a Meteorological Station Network to Provide Crop Water Requirement Information for Irrigation Management. Proceedings of the International Conference on Computer and Computing Technologies in Agriculture.

[43] Goldstein A., Fink L., Meitin A., Bohadana S., Lutenberg O., Ravid G. (2017). Applying machine learning on sensor data for irrigation recommendations: Revealing the agronomist’s tacit knowledge. Precis. Agric..

[44] Goap A., Sharma D. (2018). An IoT based smart irrigation management system using Machine learning and open source technologies. Comput. Electron. Agric..

[45] Luan Q., Fang X., Ye C., Liu Y. An integrated service system for agricultural drought monitoring and forecasting and irrigation amount forecasting. Proceedings of the 23rd International Conference on Geoinformatics, Geoinformatics 2015.

[46] Freedman D.A. (2009). Statistical Models: Theory and Practice.

[47] Iba W., Langley P., Sleeman D., Edwards P. (1992). Induction of One-Level Decision Trees. Machine Learning Proceedings 1992.

[48] Quinlan R.J. (1992). Learning with Continuous Classes. Proceedings of the 5th Australian Joint Conference on Artificial Intelligence.

[49] Wang Y., Witten I.H. Induction of model trees for predicting continuous classes. Proceedings of the Poster papers of the 9th European Conference on Machine Learning.

[50] Breiman L. (2001). Random Forests. Mach. Learn..

[51] Friedman J.H. (2000). Greedy Function Approximation: A Gradient Boosting Machine. Ann. Stat..

[52] Friedman J.H. (2002). Stochastic gradient boosting. Comput. Stat. Data Anal..

[53] Kamienski C., Soininen J.P., Taumberger M., Toscano A., Cinotti T., Dantas R., Maia R., Neto A., Furlan Ferreira F. (2019). Smart Water Management Platform: IoT-Based Precision Irrigation for Agriculture. Sensors.

[54] Kamilaris A., Gao F., Prenafeta Boldú F., Ali M.I. Agri-IoT: A Semantic Framework for Internet of Things-Enabled Smart Farming Applications. Proceedings of the 2016 IEEE 3rd World Forum on Internet of Things (WF-IoT).

[55] Pressman R. (2010). Software Engineering: A Practitioner’s Approach.

[56] Ambler S.W. (1998). Process Patterns: Building Large-Scale Systems Using Object Technology.

[57] Genuchten M.T.V. (1980). A closed-form equation for predicting the hydraulic conductivity of unsaturated soils. Soil Sci. Soc. Am. J..

[58] Torres A.B.B., Filho J.A., da Rocha A.R., Gondim R.S., de Souza J.N. Outlier detection methods and sensor data fusion for precision agriculture. Proceedings of the XXXVII Congresso da Sociedade Brasileira de Computação.

[59] Nakamura E.F., Loureiro A.A.F., Frery A.C. (2007). Information Fusion for Wireless Sensor Networks: Methods, Models, and Classifications. ACM Comput. Surv..

[60] Richards M. (2015). PyETo Implements Methods for Estimating Evapotranspiration. https://pyeto.readthedocs.io/en/latest/overview.html.

[61] FAO (1998). Crop Evapotranspiration—Guidelines for Computing Crop Water Requirements.

[62] Oracle (2019). MySQL Community Edition. https://www.mysql.com/products/community/.

[63] Czesla S. (2013). A Collection of Astronomy-Related Routines in Python. https://github.com/sczesla/PyAstronomy.

[64] Wasilak M., Amsüss C. (2012). Aiocoap—The Python CoAP Library. https://github.com/chrysn/aiocoap#aiocoap—-the-python-coap-library.

[65] Light R. (2013). Eclipse Paho MQTT Python Client. https://pypi.org/project/paho-mqtt/.

[66] Light R. (2017). Mosquitto: Server and client implementation of the MQTT protocol. J. Open Source Softw..

[67] Reitz K. (2011). Requests: HTTP for Humans. https://pypi.org/project/requests/.

[68] Richardson L. (2014). Beautiful Soup: An Screen-Scraping Library. https://pypi.org/project/beautifulsoup4/.

[69] Foundation D.S. (2013). Django—The Web Framework for Perfectionists With Deadlines. https://djangoproject.com.

[70] Encode (2011). Django Rest Framework. https://www.django-rest-framework.org/.

[71] Google (2016). Firebase—A Comprehensive App Development Platform. https://firebase.google.com/.

[72] Facebook (2018). React Native—A Framework for Building Native Apps Using React. https://facebook.github.io/react-native/.

[73] Realm (2014). Realm: Creative Mobile Apps in a Fraction Time. https://realm.io/.

[74] Invertase (2016). React Native Firebase—Simple Firebase Integration for React Native. https://rnfirebase.io/.

[75] Irrometer (2019). Irrometer Watermark 200SS Soil Moisture Sensor. https://www.irrometer.com/sensors.html#wm.

[76] Embrapa Campo Experimental do Curu of Embrapa Agroindústria Tropical. http://www.cnpat.embrapa.br/conteudo52.php.

[77] Santos H.G.d., Jacomine P.K.T., Anjos L.H.C.d., Oliveira V.A.d., Lumbreras J.F., Coelho M.R., Almeida J.A.d., Araujo filho J.C.d., Oliveira J.B.d., Cunha T.J.F. (2018). Brazilian Soil Classification System.

[78] Magalhaes R.P. (2018). Speed Prediction Applied to Dynamic Traffic Sensors and Road Networks. Ph.D. Thesis.

[79] Hall M., Frank E., Holmes G., Pfahringer B., Reutemann P., Witten I.H. (2009). The WEKA Data Mining Software: An Update. SIGKDD Explor. Newsl..

[80] Chen T., Guestrin C. (2016). XGBoost: A Scalable Tree Boosting System. Proceedings of the 22Nd ACM SIGKDD International Conference on Knowledge Discovery and Data Mining.

